# Hydrogel microarchitecture as a regulatory cue for *in vitro* odontogenic differentiation

**DOI:** 10.1590/1678-7765-2025-0607

**Published:** 2026-03-09

**Authors:** Letícia Alves Martins de Carvalho, Vitor de Toledo Stuani, Isabela Sanches Pompeo da Silva, Thayná Souza Berteli, Nicoly Gabriely Gonçalves, Diana Gabriela Soares, Ester Alves Ferreira Bordini

**Affiliations:** 1 Universidade Estadual de Campinas Instituto de Biologia Departamento de Biologia Estrutural e Funcional Campinas SP Brasil Universidade Estadual de Campinas, Instituto de Biologia, Departamento de Biologia Estrutural e Funcional, Campinas, SP, Brasil.; 2 Universidade de São Paulo Faculdade de Odontologia de Bauru, Departamento de Dentística, Endodontia e Materiais Odontológicos Bauru SP Brasil Universidade de São Paulo, Faculdade de Odontologia de Bauru, Departamento de Dentística, Endodontia e Materiais Odontológicos, Bauru, SP, Brasil.; 3 Universidade de São Paulo Faculdade de Odontologia de Ribeirão Preto Departamento de Materiais Dentários e Prótese Ribeirão Preto SP Brasil Universidade de São Paulo, Faculdade de Odontologia de Ribeirão Preto, Departamento de Materiais Dentários e Prótese, Ribeirão Preto, SP, Brasil.

**Keywords:** GelMA, Hydrogel, Dentin, Human dental pulp cells, Regenerative dentistry, Tissue engineering

## Abstract

**Objective:**

This study introduces an innovative, cost-effective, and easily reproducible strategy for engineering three-dimensional bioprinted GelMA-based scaffolds, designed with ordered macroporous and tubular architectures, and integrated microfluidic channels to advance structural and functional performance. Their geometric features were specifically designed to investigate how microarchitectural cues influence the mineralizing cell differentiation of human dental pulp cells (HDPCs).

**Methodology:**

The scaffolds were fabricated via an indirect bioprinting process using resin molds, resulting in cylindrical structures with distinct grid or honeycomb surface architectures. Biomaterials were characterized for morphology, surface topography, porosity, pore diameter, and degradability. Biological performance was evaluated by culturing HDPCs for 21 days to assess viability, proliferation, and mineralizing differentiation (ANOVA/Tukey; α=0.05).

**Results:**

Both scaffold designs exhibited interconnected porous networks, with the honeycomb configuration presenting significantly larger pores. HDPCs cultured on the scaffolds showed high viability and proliferation, with the honeycomb architecture promoting elevated ALP activity. However, the grid architecture more effectively influenced odontoblastic differentiation and mineralized matrix deposition.

**Conclusion:**

Our findings highlight the impact of biomaterial architecture on cellular behavior and reveal the potential of this novel bioprinting approach for bioactive dentin regeneration in dental tissue engineering.

## Graphical abstract

**Figure:**
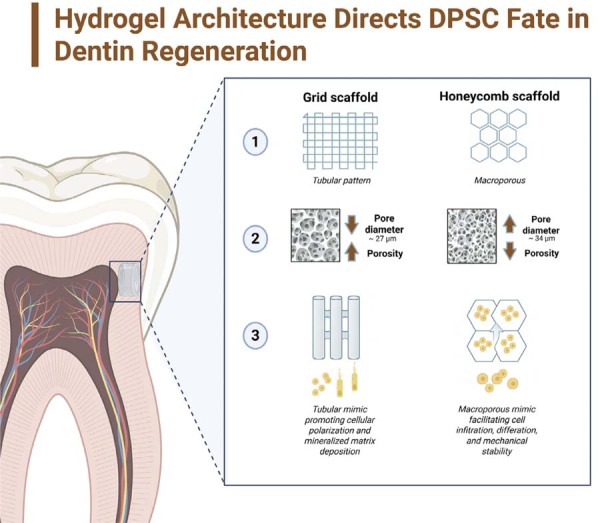

## Introduction

Teeth are complex, dynamic organs with three-dimensional structure and well-defined mechanical and biological properties.^1,2^ Dentin constitutes the bulk of the hard tissue, supporting the enamel and protecting the dental pulp.^3^ In cariogenic conditions, demineralization usually begins in enamel. Due to its highly mineralized and acellular nature and lack of regenerative capacity, enamel is particularly vulnerable to irreversible damage.^4^ Once compromised, it exposes the underlying dentin–pulp complex to the oral environment, increasing susceptibility to infections and injury.^4-6^

Under such conditions, conventional pulp-capping materials like calcium hydroxide and calcium silicate-based cements have been widely used to induce the formation of a mineralized barrier beneath the exposed pulp to preserve pulp vitality and prevent bacterial contamination.^7-10^ But several biological and mechanical limitations have been reported for these materials.^7^ Calcium hydroxide, for instance, has been shown to induce pulp inflammation, tissue necrosis, and the formation of irregular mineralized barriers when placed in direct contact with pulp tissue.^9,11,12^ Conversely, calcium silicate-based materials can induce a more predictable dentin barrier; however, they present drawbacks such as difficult handling, prolonged setting time, high cost, and moisture sensitivity.^8,13^

Seeking to overcome these limitations, and given the intrinsic regenerative potential of the dental pulp, ongoing research in restorative dentistry has increasingly focused on developing biomaterials and regenerative strategies that closely mimic the structural, biomechanical, and bioactive characteristics of native dental tissues.^14-16^ Scaffolds play a crucial role in mediating the tissue repair/regeneration process by guiding and supporting cells as they proliferate and differentiate.^17,18^ Hence their formulation from biocompatible and biodegradable polymers to ensure complete replacement by the newly deposited tissue.^19,20^

In this context, natural polymers have been widely used due to their versatility and ability to mimic the composition and microarchitecture of the dentin extracellular matrix (ECM).^18^ Such characteristics allow undifferentiated mesenchymal cells to exhibit favorable biological responses such as enhanced adhesion, proliferation, and lineage-specific differentiation when in contact with these polymers.^18,21^ Among the most used natural polymers, chitosan, alginate, and hyaluronic acid fulfill these biological requirements; however, they suffer from poor mechanical strength, limited stability, and difficulties in fabricating scaffolds with precisely controlled porous architectures.^7,22^

Thus, gelatin methacrylate (GelMA) has emerged as an advantageous alternative. This biomaterial presents a macroporous three-dimensional (3D) structure rich in collagen and RGD (arginine–glycine–aspartate) sequence which enhance cell adhesion, spreading, and proliferation on the biomaterial surface.^23^ Introducing methacrylic anhydride to the gelatin solution increases the hydrogel’s mechanical stability via the formation of unsaturated molecular bonds with the free amine groups in the polymer molecule.^22,24,25^ Additionally, this hydrogel can be polymerized after adding photoinitiators which, when activated by UV or LED light, decompose to produce free radicals that bind to the methacrylate groups, making them three-dimensionally stable at room temperature.^26^

GelMA has garnered significant attention as a versatile biomaterial, with numerous studies showing its promise not only in bone regeneration,^27-31^ but also in the engineering of vascularized mineralized tissues.^32-33^ In addition to its regenerative potential, this biomaterial also offers an effective platform for encapsulating therapeutic agents and cells.^30,34^ Moreover, its architectural configuration has emerged as a critical determinant of tissue integration and functional outcomes.^35,36^

Dentin exhibits a hierarchical microstructure composed of parallel dentinal tubules extending from the dentin-enamel junction to the pulp interface, forming an intricate network of microchannels. Replicating these morphological features in biomaterial design is essential to support nutrient transport and cellular communication.^37^ Although contemporary additive manufacturing techniques allow precise control over scaffold architecture, their broader clinical and research applicability remains limited by high equipment costs and technical complexity.^38^ Seeking to overcome these practical and economic barriers, we developed a novel, low-cost fabrication strategy that employs geometrically negative molds for GelMA hydrogel casting. This method enables producing tubular scaffold architectures that closely resemble native dentin morphology, providing a cost-effective, robust, and reproducible alternative for engineering biomimetic scaffolds.

To assess the versatility and regenerative potential of this approach, we designed grid and honeycomb scaffold geometries and examined how their microarchitectural features influence HDPC adhesion, proliferation, and differentiation toward an odontoblastic phenotype. These insights advance the rational design of architected biomaterials and establish a promising biomimetic platform for regenerating mineralized tissues. To the best of our knowledge, this is the first study to fabricate biomimetic dentin-like tubular scaffolds using a low-cost negative-mold strategy compatible with conventional laboratory resources.

## Methodology

### Synthesis of gelatin methacryloyl hydrogel

Gelatin methacryloyl (GelMA) used in this study was synthesized following the same validated protocol previously established by our group^39^ and fully characterized by Ribeiro, et al.^40^ (2020), including confirmation of its elemental and structural composition. Type A porcine skin gelatin (Sigma-Aldrich) was dissolved in phosphate-buffered saline (PBS) to obtain a 10% (w/v) solution under magnetic stirring at 50 °C. After complete homogenization, 8 mL of methacrylic anhydride (Sigma-Aldrich) was added dropwise while maintaining the temperature at 50 °C, and the reaction was allowed to proceed for 1 h to ensure efficient methacrylation. The reaction mixture was then diluted with 100 mL of preheated PBS and transferred into dialysis membranes (Spectro/Pot, MWCO 12–14 kDa, Fisher Scientific). Dialysis was performed against deionized water at 50 °C for 5 days, with three water changes per day to remove unreacted monomers and low-molecular-weight impurities. Following this, the solution was filtered through a 0.22 µm membrane, dispensed into tubes, frozen at −80 °C, and lyophilized under vacuum (150×10⁻^3^ mbar) at −52 °C for 5 days. The resulting GelMA foam was stored at −20 °C until use. For photo-crosslinking, GelMA solutions were prepared with 0.075% lithium phenyl-2,4,6-trimethylbenzoylphosphinate (LAP) as the photoinitiator and polymerized under LED light.

### Indirect printing technique

Architectural molds incorporating grid and honeycomb geometries were digitally designed on Meshmixer software (Autodesk Inc.). The grid design featured quadrangular channels (500 μm width × 500 μm length × 3 mm height), whereas the honeycomb design consisted of hexagonal channels (500 μm diameter × 3 mm height). A 1 mm-thick removable platform was integrated at the mold base to facilitate hydrogel retrieval. The digital models were processed using ChiTuBox (CBD-Tech) slicing software and printed with Anycubic Gray resin on a Anycubic Photon Mono SE printer (Anycubic, Shenzhen, China). After printing, the molds were washed with isopropyl alcohol for 10 minutes using a Wash and Cure device (Anycubic). They were then post-cured under UV light for an additional 10 minutes on the same device. Afterward, the molds were disinfected with 70% ethanol, rinsed with distilled water, and UV sterilized for 30 minutes.

**Figure 1:**
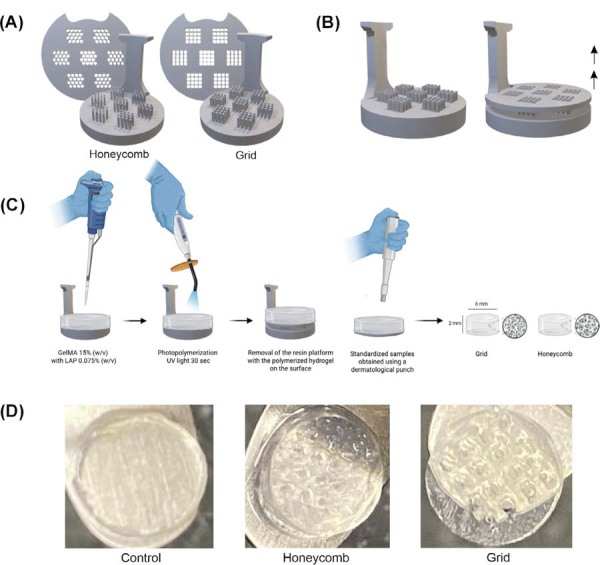
Fabrication process of architected GelMA hydrogels. (A) Digital design of the negative molds featuring honeycomb and grid architectures. (B) Illustration of the removable platform at the base of the molds, designed to facilitate the retrieval of the polymerized hydrogel. (C) Schematic illustration of the indirect printing technique: the GelMA (15% w/v) solution with LAP photoinitiator (0.075% w/v) is cast into the mold, followed by photopolymerization with UV light for 30 seconds. After removal of the resin platform, the samples are standardized to a 6 mm diameter and 2 mm thickness using a biopsy punch. (D) Macroscopic images of the resulting hydrogels: control (non-patterned), honeycomb, and grid.

Hydrogel solution was prepared by sterilizing lyophilized GelMA (15% w/v) under UV light and subsequently dissolving it in sterile PBS containing the photoinitiator. The solution was thoroughly homogenized and centrifuged at 5,000 rpm for 5 minutes to remove air bubbles. One milliliter of the mixture was transferred into each mold and photopolymerized for 30 seconds on each side using an LED curing unit with a wavelength range of 285–515 nm and an irradiance of 1200 mW/cm^2^ (Bluephase N, Ivoclar Vivadent, Buffalo, NY, USA). Samples were standardized to a 6 mm diameter and 2 mm thickness using a biopsy punch (Kolplast, Itupeva, SP, Brazil), ensuring the preservation of their architectural features. Control samples were prepared using a 15% GelMA solution without architectural patterns, then photopolymerized and punched to identical dimensions (Figure 1).

### Morphological and physical characterization of 3D indirectly printed GelMA hydrogels

#### Surface architecture and topography evaluation

Scanning electron microscopy (SEM; PSEM Expres, Aspex Altmann, Delmont, PA, USA) analyses were performed at an acceleration voltage of 15 kV after printing the hydrogels. Following printing, the scaffolds (n=4) were frozen at −80 °C, freeze-dried overnight at −56 °C (150 × 10⁻^3^ mbar; FreeZone, Labconco Corporation, Kansas City, MO, USA), mounted on metallic stubs, and sputter-coated with gold. Lyophilization was necessary to enable high-vacuum imaging and validate the fidelity of the macro-architectural features transferred from the molds, although it inherently dehydrates the intrinsic polymeric mesh.

Pore diameter (µm) and porosity (%) were quantified on ImageJ software (National Institutes of Health, Bethesda, MD, USA). For pore size analysis, 100 pores were randomly selected from a total of 30 SEM images per material, and measurements were obtained using the Straight-Line tool. Porosity (%) was determined using the Threshold tool in ImageJ, which allows quantification of the proportion of pore area relative to the total image area.^41^ These quantitative assessments guided the selection of the printing parameters that yielded the most uniform and structurally consistent architectures based on pore morphology, porosity distribution, and internal structural homogeneity.

## Hydrolytic stability and mass loss analysis of hydrogels

Hydrolytic stability of the biomaterials with distinct architectural patterns was evaluated by monitoring mass variation under physiologically relevant conditions. Standardized samples (n=6) were immersed in 1.5 mL of phosphate-buffered saline (PBS) at 37 °C for 24 hours to reach thermodynamic equilibrium (maximum swelling). Excess surface moisture was removed by gently blotting each side with absorbent paper, and the initial swollen weight (Pi) was recorded using an analytical balance (Mettler Toledo XS105 DualRange). Samples were then maintained in PBS at 37 °C, with the solution being replaced weekly throughout the 28-day period. Mass measurements were performed on days 1, 3, 7, 14, 21, and 28. Degradability (DG) and mass variation were calculated as DG(%)=[(Pu−Pi)/Pi]×100 , in which Pu represents the wet weight at each time point.

## Biological characterization of hydrogels with different architectures

### Cultivation of undifferentiated human dental pulp mesenchymal cells (HDPCs)

HDPCs were obtained from Lonza Bioscience (Catalog #: PT-5025, Basel, Switzerland). Cells were expanded and cultivated in complete α-MEM culture medium (Minimum Essential Medium Eagle Alpha, supplemented with 10% fetal bovine serum – FBS, L-glutamine, and 1% penicillin-streptomycin; Gibco^®^, Invitrogen, Carlsbad, CA, USA), according to the manufacturer’s recommendations.

## Experimental design

The scaffolds (6 mm diameter × 2 mm thickness) were sterilized under UV light for 30 minutes on each side within a laminar flow hood. Subsequently, they were placed in 48-well plates containing 1 mL of complete α-MEM culture medium and incubated overnight. HDPCs were seeded at a density of 1x10⁵ cells per scaffold using a single droplet technique to prevent dispersal beyond the biomaterial boundaries. The cell-seeded constructs were incubated at 37°C with 5% CO₂ for 30 minutes to facilitate initial cell attachment, after which 1 mL of culture medium was added. Subsequently, the cell-hydrogel constructs were cultured for up to 21 days.

For differentiation studies (ALP activity and Alizarin red assays), cells were cultivated in osteogenic medium (complete α-MEM supplemented with 50 μg/mL ascorbic acid and 5 mM β-glycerophosphate). Each analysis included six samples per group per time point, with three independent experimental replicates performed.

## Cell viability (Live/Dead)

After 21 days of culture (n=3), the scaffolds were washed in PBS and incubated for 20 minutes with α-MEM supplemented with Calcein AM and Ethidium Homodimer-1 (Live/Dead cell viability/cytotoxicity kit; Invitrogen, San Francisco, CA, USA) at a concentration of 1:1000. Afterwards, the samples were washed thrice with PBS. Samples were then positioned under a glass coverslip for assessment of viable and non-viable cells on the material surface. Cell viability was analyzed using a fluorescence microscope (DM5500B, Leica Microsystems, Germany).

## Cell proliferation

After 3, 7, 14, and 21 days of culture, HDPCs were assessed for cell proliferation using the Alamar Blue assay. For this, a solution of α-MEM without FBS containing 10% Alamar Blue reagent (Life-Technologies) was prepared in a 10:1 ratio. This solution was incubated with the constructs (HDPCs/hydrogels) for 4 hours at 37°C and 5% CO₂. Following the incubation period, the supernatant was transferred to a 96-well plate. Fluorescence was measured at 540 nm excitation and 590 nm emission using a Synergy H1 plate reader (Biotek, Winooski, USA). The mean fluorescence value obtained from HDPCs cultured directly on unpatterned GelMA (control) was considered to represent 100% cell proliferation. Proliferation in the experimental groups was then expressed as a percentage relative to this reference value (n=6).

## ALP activity

Alkaline phosphatase (ALP) activity was evaluated in indirectly printed hydrogels (n=6) at days 14 and 21 using a commercial endpoint ALP assay kit (Labtest Diagnóstico S.A., Lagoa Santa, MG, Brazil), which is based on the thymolphthalein monophosphate method. At each time point, samples were incubated with 0.1% sodium lauryl sulfate at room temperature for 40 minutes. Subsequently, the hydrogels were mechanically disrupted in a lysis buffer and centrifuged at 4,000 rpm for 10 minutes to obtain the supernatant, which was then transferred to vials containing thymolphthalein monophosphate substrate (22 mmol/L, pH 10.1; Labtest Diagnostica S.A.) and incubated at 37 °C for 15 minutes. After incubation, a colorimetric reagent consisting of 94 mmol/L sodium carbonate and 250 mmol/L sodium hydroxide (Labtest Diagnostica S.A.) was added, and absorbance was measured at 590 nm (Synergy H1).

For normalization, total protein content was determined using Lowry’s method. The supernatant was incubated with Lowry reagent for 20 minutes at room temperature, followed by the addition of Folin–Ciocalteu phenol reagent (Sigma-Aldrich) and further incubation for 30 minutes under the same conditions. Absorbance was measured at 655 nm (Synergy H1). Final alkaline phosphatase (ALP) activity was determined by calculating the ratio of ALP concentration to total protein content, with both parameters quantified based on their respective standard curves.

## Mineralized matrix deposition

Alizarin Red staining was performed on days 14 and 21 of HDPC culture on hydrogels with distinct architectural patterns. The samples were fixed in 70% ethanol at 4 °C for 1 hour, thereafter rinsed with deionized water, and subsequently incubated with Alizarin Red solution (40 mM, pH 4.2; Sigma Chemical, Sigma-Aldrich) for 15 minutes under agitation. Following incubation, the hydrogels were thoroughly washed with deionized water and then incubated with a 10 mM cetylpyridinium chloride solution (pH 7.0; Sigma Chemical, Sigma-Aldrich) to dissolve the mineralized nodules. Finally, the absorbance of the resulting solution was measured at 560 nm (Synergy H1). Hydrogels with grid and honeycomb surface architectures, cultured in osteogenic medium without cells, were used as background controls (n=6).

## Statistical analysis

Data distribution normality and homoscedasticity were assessed by Shapiro–Wilk and Levene tests, respectively. Statistical analyses were performed on GraphPad Prism 9 (San Diego, CA, USA). One-way ANOVA was used exclusively for single-factor comparisons. For longitudinal datasets involving repeated measurements across time and scaffold architecture, a two-way ANOVA evaluated the main effects of time and architecture, as well as their interaction. Tukey’s post hoc test was used for multiple comparisons. Statistical significance was set at p<0.05.

## Results

### Microstructural characterization and degradation profile of gelma hydrogels

**Figure 2:**
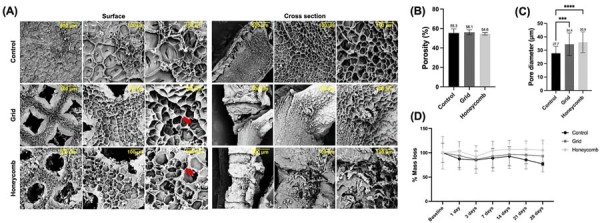
Microstructural characterization and in vitro mass stability of hydrogel with distinct architectures. (A) Scanning Electron Microscopy (SEM) images of the surface and cross section of the control (non-patterned), grid, and honeycomb hydrogels. Micrographs were taken at 100x, 250x and 500x magnifications. Scale bars = 500 µm and 100 µm. (B) Bar graph showing the mean and standard deviations of % porosity and (C) pore size (One way ANOVA/Tukey's test. Significance levels: ***p < 0.0005, ****p < 0.0001). (C) Graph of the degradation profile, showing the percentage of remaining hydrogel mass over 28 days of incubation. Data points represent the mean ± standard deviation of independent samples (Two-way ANOVA/Tukey's test; p<0.05; n=6).

SEM analysis assessed the architectural fidelity of the hydrogels produced using indirect mold technique. The micrographs revealed distinct structural patterns among the groups (Figure 2A). Control group, built without a guiding mold, exhibited a heterogeneous porous matrix with irregular pore shapes and variable diameters. In contrast, the grid and honeycomb hydrogels displayed organized surface macro-architectures that accurately reproduced the geometry of the negative molds. Grid samples showed a consistent and uniform distribution of tubular channels throughout the matrix. Conversely, honeycomb hydrogels presented large surface pores matching the designed pattern, whereas their internal regions displayed a less organized morphology with reduced pore density. Notably, although the intrinsic microporosity of the polymeric network may be altered by lyophilization, the engineered macro-features remained distinct and well preserved.

Quantitative analysis revealed that total porosity was comparable across all groups (Figure 2B), with no statistically significant differences detected (p>0.05). Pore diameter presented significant differences (Figure 2C), with honeycomb hydrogels exhibiting a markedly larger mean pore size compared with both grid and control samples (p<0.05).

Regarding stability, all groups maintained their three-dimensional structural integrity after 28 days of incubation (Figure 2D). Remaining wet mass analysis revealed distinct degradation profiles among the hydrogels. The honeycomb group showed the highest stability, maintaining 100% of its initial mass throughout the experimental period. On day 21, the control and grid groups retained 85% and 95% of their initial mass, respectively. By day 28, the control group exhibited a more pronounced reduction, retaining 77% of its initial mass which was significantly lower than that of the honeycomb group (p<0.01).

### Cell viability and proliferation

**Figure 3:**
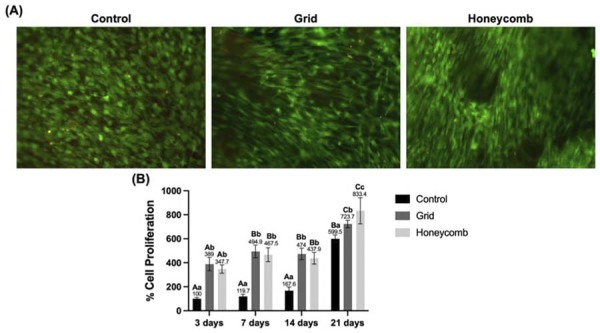
Cell viability and metabolism in GelMA hydrogels with distinct architectures. (A) Live/Dead cell viability assay micrographs after 21 days of culture, showing live (green) and dead (red) cells on the control, grid, and honeycomb hydrogels. (B) Graph quantifying cellular proliferation (% of control at the initial time point) on the hydrogels over 21 days. Bars represent the mean ± standard deviation. Different letters indicate statistically significant differences between groups at each time point (uppercase letters) and over time within each group (lowercase letters) (Two-way ANOVA/Tukey´s; p<0.05; n=6).

Cell viability of the hydrogels was confirmed by Live/Dead assay after 21 days of culture (Figure 3A). Qualitative analysis revealed a predominance of live cells (green staining) across all groups (control, grid, and honeycomb), indicating good biocompatibility of the different hydrogel architectures. Only a few dead cells (red staining) were observed in all groups.

Cell metabolic activity was quantified over the 21-day period (Figure 3B), showing successful HDPCs adhesion and proliferation on all hydrogel architectural surfaces. All groups exhibited progressive increase in metabolic activity over the experimental period. At days 3, 7, and 14, HDPCs cultured on both hydrogel architectures showed higher metabolic activity than the control (3 days: 100.0%±9.94, 7 days: 119.7%±15.9, 14 days: 167.7±25.9; n=6). By day 21, the honeycomb architecture reached 833.4 ± 94.3, which was significantly higher than the grid (723.7±28.2) and control (599.5±28.1) groups (two-way ANOVA, Tukey’s post-hoc test, p<0.05). Within-group analyses revealed a significant increase in metabolic activity from day 3 to day 21 for all three conditions (p<0.05).

### Mineralizing lineage differentiation and mineralized matrix deposition

**Figure 4:**
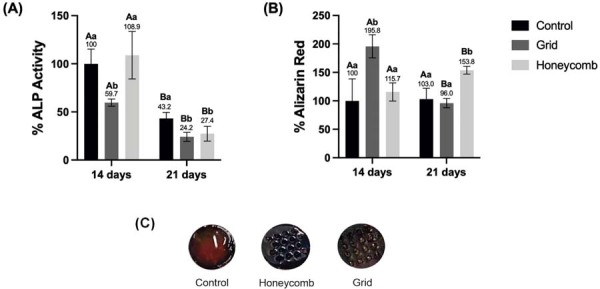
Analysis of odontogenic differentiation on GelMA hydrogels. (A) Quantification of Alkaline Phosphatase activity, an early marker of odontogenesis, at days 14 and 21. (B) Quantification of calcium deposition by Alizarin Red staining, a mid-stage indicator of matrix mineralization, at days 14 and 21. Data were normalized to the Control group at day 14. Bars represent the mean ± standard deviation. Different uppercase letters indicate a statistically significant difference (p<0.05) over time within the same group. Different lowercase letters indicate a statistically significant difference (p<0.05) between groups at the same time point (Two-way ANOVA/Tukey´s; p<0.05; n=6).

Early mineralizing differentiation was assessed by alkaline phosphatase (ALP) activity (Figure 4A). On day 14, the honeycomb group showed a slight increase in ALP activity (8.9%), followed by a decline in activity at day 21, reflecting the typical progression of cellular maturation. Cells cultured on the grid-architectured hydrogel exhibited lower ALP activity at both day 14 (59.7%) and day 21 (50.9%) compared with control (p<0.05).

Mineralized matrix deposition quantification revealed significant differences among the groups and over time (Figure 4B). At day 14, the grid group exhibited a significantly higher level of calcium deposition (195.8%) compared with both the honeycomb and control groups (p<0.05). A marked increase in mineralization was observed in the honeycomb group (153.8%) from day 14 to day 21 (p<0.05), whereas the grid group showed a reduction over the same period (96.0%). No statistically significant changes in calcium deposition were detected in the control group at any time point.

## Discussion

Dentin tissue regeneration remains a persistent focus of research due to the substantial challenges associated with developing suitable materials for clinical application. Ideally, such materials should replicate the characteristics of the compromised dentin extracellular matrix, thereby promoting the migration and adhesion of undifferentiated mesenchymal cells from the dental pulp to the injury site.^42^ These cells can then differentiate into odontoblast-like cells, contributing to restore physiological tissue functions by forming reparative dentin.^43-45^

In this context, numerous studies have explored the development of biomaterials combined with various nanoparticles and signaling molecules to engineer biomimetic scaffolds that address the limitations of currently available materials.^15,46-49^ But several challenges persist, including the high costs associated with manufacturing processes, the controlled release of therapeutically relevant drug dosages, and the preservation of molecular integrity until clinical application.^50^

As such, we hypothesized that the precise control of hydrogel microarchitecture constitutes a critical determinant of cellular behavior and regenerative outcomes in engineering functional substitutes for the dentin-pulp complex. To test this hypothesis, we designed two distinct architectural configurations, each strategically tailored to elicit a specific biological response. Specifically, a grid-like (tubular) pattern to emulate the odontoblast-dentin interface and promote cell polarization, and a honeycomb (macroporous) pattern to facilitate cell infiltration and enhance cellular viability. Our findings support this hypothesis, showing that hydrogel architecture can be purposefully designed to orchestrate distinct yet complementary tissue regeneration phases.

Indirect fabrication strategy was adopted due to well-recognized limitations of direct hydrogel bioprinting for producing fine-scale, dentin-like architectures. Low-viscosity polymer solutions like GelMA often exhibit insufficient shape fidelity prior to crosslinking, rendering it challenging to generate small, ordered, and well-defined pore geometries using direct extrusion methods.^51^ Moreover, pure GelMA lacks the mechanical stability required to preserve its printed form immediately after deposition, often requiring the addition of rheology-modifying components to improve structural integrity and printability.^51^

Converszely, the use of high-resolution, 3D-printed negative molds enables precise control over architectural features, including the formation of tubular and macroporous structures that closely mimic native dentin. This approach is also highly reproducible, cost-effective, and compatible with laboratory-scale workflows, allowing the rapid production of multiple scaffolds with consistent geometry. Together, these advantages underscore the suitability of indirect molding as a robust and accessible alternative for engineering biomimetic scaffolds for dentin regeneration.

Notably, the GelMA formulation used here has been thoroughly characterized in prior work,^40^ and the standardized synthesis protocol employed in our laboratory consistently yields reproducible polymer compositions. Thus, although additional elemental confirmation was not repeated in this study, this limitation does not affect the interpretation of the biological outcomes reported.

Biological analyses revealed that honeycomb architecture proved superior for promoting early-stage cell functions and ensuring material stability. We attribute this success to its biomimetic hexagonal geometry, which optimizes the surface-to-volume ratio and ensures uniform stress distribution throughout the material.^52,53^ Direct evidence from our study includes the significantly lower mass loss of honeycomb hydrogels during degradation assays, indicating superior structural integrity. Moreover, the enhanced ALP activity in this group suggests that architecture creates a microenvironment highly conducive to early mineralizing lineage differentiation.

These findings are consistent with previously published studies showing that biomaterial surface topography customization directly influences the behavior and differentiation of undifferentiated mesenchymal cells toward specific phenotypes.^46,47,50^ Notably, Olivares, et al.^54^ (2009) further established that the geometry of the biomaterial modulates mechanotransduction pathways that govern stem cell fate, thereby supporting the efficacy of the honeycomb pattern in facilitating both colonization and early differentiation of HDPCs. This process is initiated at the molecular level when geometric cues are sensed by fibronectin-bound integrin receptors, which organize the formation of mature Focal Adhesions (FAs).^55^ These mature FAs, in turn, enhance the clustering of cytoskeletal stress fibers, generating concentrated contractile forces. This force is then transmitted to the nucleus, controlling the nuclear translocation of key mechanosensors like YAP/TAZ, which ultimately modulates the switch in cell commitment towards odontogenesis.^55^

We observed a clear functional dichotomy between the grid and honeycomb architectures in the study results, suggesting that each configuration could be strategically tailored to address distinct dentin regeneration stages. The honeycomb architecture, characterized by its open and highly interconnected network (55.3%), provides an optimal microenvironment for nutrient diffusion and intercellular signaling, thereby effectively supporting cell proliferation and early-stage differentiation.^40^ This concept is corroborated by Takabatake, et al.^52^ (2020), who established that the geometric design of honeycomb scaffolds is not merely structural but bioactive. They revealed that specific pore configurations in honeycomb architectures effectively recapitulate the native extracellular matrix microenvironment, facilitating the necessary interactions between progenitor cells and their surroundings to support tissue genesis.

In contrast, the enhanced performance of the grid architecture during the later regeneration phases, despite comparable early-stage ALP activity, highlights this precise functional specialization. While the honeycomb’s geometry is a potent inducer of early mineralizing commitment, the grid’s architecture provides the distinct cues necessary for mid-stage functional maturation. Its tubular structure closely replicates the native dentin organization, imposing contact guidance on the cells and forces an elongated and polarized morphology, which is a well-established prerequisite for the mature, secretory phenotype of odontoblasts responsible for structured mineral deposition.^56^ This architectural specialization highlights a promising approach for developing multi-phase or layered biomaterials designed to achieve comprehensive and temporally coordinated tissue regeneration.

This late-stage functional specialization is further supported and amplified by the distinct physical and biochemical properties of the grid architecture. Firstly, the grid’s high structural integrity, as evinced in the SEM images and consistent with the high compressive strength reported by Schmidleithner, et al.^57^ (2019), creates a mechanically competent microenvironment. This stiffness is itself a well-established promoter of cell differentiation and mineral deposition.^58^ Secondly, beyond the bulk mechanics, its specific microtopography may enhance the material’s surface reactivity. This could establish more effective nucleation sites for calcified deposits, analogous to the behavior of bioactive ceramics. Additionally, as discussed by Gurucharan, et al.^7^(2023), its tubular structure, designed to mimic native dentin, may also play a biological role by optimizing the sequestration or concentration of endogenous growth factors released by the cells, such as TGF-β1 and Col-I, which are known to potently increase mineralized activity.

The relevance of scaffold architecture in modulating mineralizing outcomes is further supported by Lee, et al.^59^ (2012), who evinced that poly(ε-caprolactone) nanofibrous meshes mimicking the dentin extracellular matrix enhanced DPSC adhesion, proliferation, and differentiation. Their study showed that the scaffold acted as a hydrophobic barrier that reduced the cytotoxic effects of hydroxyl ions released by MTA while synergistically promoting mineralizing cell differentiation when used in combination with the cement. *In vitro*, the composite system increased DSPP expression, ALP activity, and calcium deposition; *in vivo,* it stimulated the formation of a thick, well-organized dentin bridge populated by polarized odontoblast-like cells.

Moreover, the relevance of scaffold microarchitecture in directing cell fate is further supported by studies showing that subtle variations in porosity and pore geometry can modulate osteogenic and odontogenic outcomes.^52,54,55,60^ Hayashi and Ishikawa^60^ (2020) reported that grid-shaped scaffolds with 54.9% porosity promoted significantly greater bone formation after four weeks *in vivo*, whereas macroporous (pore volume 0.15 cm^3^ g⁻^1^; porosity 58.9%) and nanoporous structures (pore volume 0.07 cm^3^ g⁻^1^; porosity 50.8%) negatively affected bone deposition even after twelve weeks. Similarly, Takabatake, et al.^52^ (2020) observed that honeycomb architectures with pore diameters of 75 µm and 300 µm enhanced cytoskeletal organization, cell adhesion, and early mineralizing differentiation, with the 300 µm pores supporting tissue formation that closely resembled dentin. These findings align with our results and reinforce the concept that engineered microarchitectures can be strategically designed to modulate cell behavior at the dentin–pulp interface, creating a microenvironment conducive to mineralized tissue formation and ultimately enhancing the regenerative potential of the material.

Importantly, SEM imaging presents inherent limitations when used to characterize hydrogels in their native, hydrated conformation.^61^ The freezing and lyophilization steps required for sample preparation can induce irreversible alterations in the original microarchitecture, including changes in pore size, morphology, and spatial distribution resulting from structural collapse or deformation during dehydration.^61-63^ Despite these artifacts, SEM remains a well-established and widely adopted technique for pore assessment due to its ability to provide high-resolution visualization of scaffold morphology and surface topology, offering structural insights that are difficult to obtain using other methodologies.^63^

An ideal biomaterial for tissue regeneration must maintain a balance between structural integrity and a degradation profile compatible with new tissue formation.^2^ In the remaining mass analysis, the honeycomb architecture showed remarkable stability, maintaining its initial mass throughout the 28-day period. Conversely, both the grid and control groups exhibited a slight reduction in remaining mass beginning at day 21. We argue that the structural integrity and uniform stress distribution inherent to the honeycomb architecture not only enhance its mechanical performance but also contribute to its resistance against hydrolytic degradation. These findings align with previous reports revealing enhanced mass retention and reduced degradation rates in specific hydrogel formulations under enzyme-free conditions.^30^

Regarding this assessment, we acknowledge that our analysis monitored hydrogel swollen weight in a physiological environment, rather than dry polymer mass. We attribute the mass variations observed, particularly in the initial phase, to thermodynamic equilibration (hydrogel deswelling) and structural adjustments rather than to immediate polymer erosion. Although dry weight evaluation is the standard method for quantifying polymer degradation, our approach provides a relevant translational parameter: the macroscopic volume maintenance of the construct. In a clinical scenario, the scaffold’s ability to preserve its dimensions and remain adequately hydrated is essential to ensure the defect remains filled during the early stages of healing.

Given that *in vivo* dentin barrier formation can occur within approximately four weeks,^64^ the stability observed in our constructs, particularly the honeycomb architecture, appears well-suited to support the entire course of dentin regeneration.

## Conclusion

We developed and validated an innovative and low-cost fabrication strategy based on indirect 3D-printed molds to produce architected hydrogels. Our primary contribution lies in bridging the gap between advanced scaffold design and its accessible, cost-effective implementation. Our findings show that controlled architectural fabrication enables the functional specialization of biomaterials to support distinct dentin regeneration phases. Combining features like the honeycomb design, which enhances early cell colonization, with the grid design, which promotes late-stage mineralization, may represent an effective strategy to improve regenerative dental therapies. This architectural control also enabled engineering biomaterials with enhanced stability against degradation without compromising their interconnected porous network. Future work should directly test this multi-stage hypothesis, potentially via hybrid or layered hydrogels, incorporate dentinogenic growth factors, assess odontogenic-specific markers to further confirm the applicability of these scaffolds for dentin regeneration, and to validate these promising results in clinically relevant *in vivo* models. This approach represents a significant step toward developing customized and more effective biomaterials for dental tissue engineering.
